# Supplementary material for: The effects of chikungunya virus infection on people living with HIV during the 2014 Martinique outbreak
Source: PLoS One. 2020 Jun 5;15(6):e0234267. doi: 10.1371/journal.pone.0234267 (PMC7274814; doi:10.1371/journal.pone.0234267)
Supplement: S1 Table — (DOCX) [file pone.0234267.s002.docx]

**S1 Table: Age and gender repartitions of the Martinique population and the PLHIV during the 2014 outbreak**

|  | Male | | | | Female | | | |
| --- | --- | --- | --- | --- | --- | --- | --- | --- |
|  | Population | | PLHIV | | Population | | PLHIV | |
| Age | N | % | N | % | N | % | N | % |
| 18-19 | 5103 | 1.71 | 2 | 0.20 | 4650 | 1.56 | 1 | 0.10 |
| 20-29 | 18435 | 6.19 | 46 | 4.59 | 20061 | 6.74 | 35 | 3.49 |
| 30-39 | 17193 | 5.78 | 72 | 7.18 | 23707 | 7.96 | 62 | 6.18 |
| 40-49 | 26986 | 9.07 | 176 | 17.55 | 33931 | 11.40 | 112 | 11.17 |
| 50-59 | 27054 | 9.09 | 214 | 21.34 | 31492 | 10.58 | 91 | 9.07 |
| 60-69 | 19486 | 6.55 | 85 | 8.47 | 22922 | 7.70 | 46 | 4.59 |
| 70-79 | 12530 | 4.21 | 32 | 3.19 | 15618 | 5.25 | 20 | 1.99 |
| 80-89 | 5764 | 1.94 | 7 | 0.70 | 8961 | 3.01 | 2 | 0.20 |
| ≥ 90 | 1083 | 0.36 | 0 | 0.00 | 2703 | 0.91 | 0 | 0.00 |
| Total | 133636 | 44,89 | 634 | 63,22 | 164045 | 55,11 | 369 | 36,79 |
