# Supplementary material for: The effects of chikungunya virus infection on people living with HIV during the 2014 Martinique outbreak
Source: PLoS One. 2020 Jun 5;15(6):e0234267. doi: 10.1371/journal.pone.0234267 (PMC7274814; doi:10.1371/journal.pone.0234267)
Supplement: Attachment — Submitted filename: response to reviewers.docx [file pone.0234267.s003.docx]

Dear Editor

Thank you for the time you and the reviewer took to assess our manuscript. We did our best to answer the wanted revisions.

**Editor queries**

1. It should be important to highlight the current situation of HIV prevalence in the study period

Answer: Some details have been added in the Background paragraph

2. Please ensure that your manuscript meets PLOS ONE's style requirements, including those for file naming.

Answer: We did our best

2. Please provide additional details regarding participant consent. In the ethics statement in the Methods and online submission information, please ensure that you have specified whether consent was suitably informed.

Answer: Details have been added in the data collection and ethics statement part

3. Please include additional information regarding the survey or questionnaire used in the study and ensure that you have provided sufficient details that others could replicate the analyses. If you developed and/or translated a questionnaire as part of this study and it is not under a copyright license more restrictive than Creative Commons Attribution (CC-BY), please include a copy, in both the original language and English, as Supporting Information.

Answer: As we understand that the formulation led to misunderstanding, I rephrased the sentence, line 58 of the Manuscript with track changes

4. Please ensure that you refer to Figure 2 in your text as, if accepted, production will need this reference to link the reader to the figure.

Answer: Edited

**Reviewer’s queries**

1) the numbering system used needs to be adjusted to the US numbering format.

Answer: I did my best
2) Line 121, please describe that the virus load needed is the HIV.

Answer: Edited
3) in describing population, please include the upper and lower limit of the age group not just the median age.

Answer: I did, although quartiles may be more informative, thus I propose to keep both

5) A general description for the level of CHIKV infection in Martinique during the outbreak would be useful.

Answer: As stated in the Introduction, CHIKV infection affected about 40% of the island population

6) can you please include a timeline for when the questionnaires of patients were taken post the outbreak? this would help assess how much of recall bias is to be expected

Answer: Precision has been added (L123 and 124 of the revised manuscript with track changes) regarding the time elapsed between clinical manifestations, assessment of symptoms and biological confirmation for the overall population.
